# Follicular Unit Extraction Hair Transplant

**DOI:** 10.4103/0974-2077.69015

**Published:** 2010

**Authors:** Aman Dua, Kapil Dua

**Affiliations:** *Department of Dermatology, Venereology and Leprology, AK Clinics, Ludhiana, Punjab, India*; 1 *Department of ENT, Dayanand Medical College and Hospital, Ludhiana, Punjab, India*

**Keywords:** Follicular unit extraction, FOX test, hair transplant, sutureless

## Abstract

Hair transplantation has come a long way from the days of Punch Hair Transplant by Dr. Orentreich in 1950s to Follicular Unit Hair Transplant (FUT) of 1990s and the very recent Follicular Unit Extraction (FUE) technique. With the advent of FUE, the dream of ‘no visible scarring’ in the donor area is now looking like a possibility. In FUE, the grafts are extracted as individual follicular units in a two-step or three-step technique whereas the method of implantation remains the same as in the traditional FUT. The addition of latest automated FUE technique seeks to overcome some of the limitations in this relatively new technique and it is now possible to achieve more than a thousand grafts in one day in trained hands. This article reviews the methodology, limitations and advantages of FUE hair transplant.

## INTRODUCTION

Modern hair transplantation was introduced in the 1950s by Dr. Orentreich.[1] He started with the help of 4 mm punches. Then the concept of mini and micrografting,[2,3] and later in 1990s the Follicular Unit Hair Transplantation (FUT)[4] took over. With FUT, transplantation of hair in naturally occurring individual follicular units was established.[5] In these methods, donor harvesting was done by single strip method with elliptical excision of donor, followed by suturing. The significant disadvantage of single strip harvesting was the resultant linear donor scar. Though it is possible to provide a very fine linear scar with the newly described trichophytic closure,[6,7] it does pose cosmetic problems for many patients particularly those who wish to wear short hair.[8,9] Bernstein and Rassman[10] started developing the FOX procedure, heralding a new surgical hair restoration procedure without strip harvesting. The FOX procedure, also known as FUE (Follicular Unit Extraction), FUSE (Follicular Unit Separation Extraction) method,[11] Wood’s technique,[12] FU Isolation method[13] is fast becoming an alternative method of extraction of grafts as follicular units in selected cases. While there are many limitations to this new technique, several new developments are taking place to overcome the limitations of number of grafts in one session of FUE.

This article presents a review of different aspects of FUE such as, the prerequisites of doing FUE hair transplant, indications and contraindications, procedure, limitations and the latest advancements in the field of FUE.

## PRINCIPLE OF FOLLICULAR UNIT EXTRACTION

**Figure 1:**
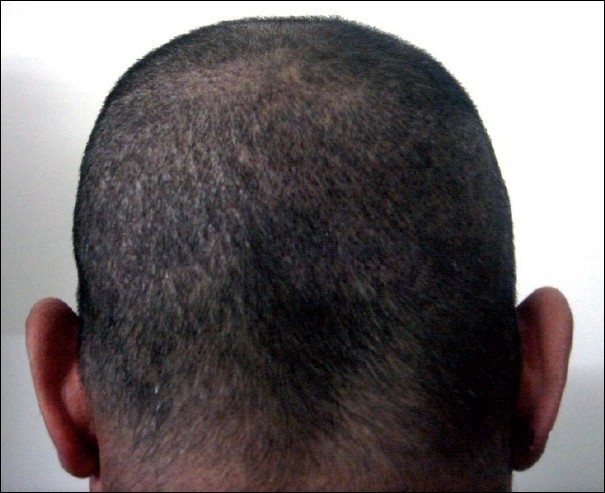
Donor area after FUE hair transplant after 7 days

In FUE, the extraction of intact follicular unit is dependent on the principle that the area of attachment of arrector muscle to the follicular unit is the tightest zone. Once this is made loose and separated from the surrounding dermis, the inferior segment can be extracted easily. Because the follicular unit is narrowest at the surface, one needs to use small micropunches of size 0.6–0.8 mm and therefore the resulting scar is too small to be recognised [Figure 1].

The main anatomical limitation of the technique is that it is not possible to identify the bulge of the hair from outside and hence the procedure is blind. Also, since the hairs with intact unit splay at the lower end and diverge in different directions, the process of extraction can result in a higher transection rate. The procedure is also slow as each unit has to be pulled out slowly. However, with experience, the hand eye coordination and speed of the surgeon, transection rate can be improved.

## PREREQUISITES FOR DOING FOLLICULAR UNIT EXTRACTION

Following are the prerequisites for doing FUE:[14]

- Adequate experience and training of the surgeon
- Excellent lighting
- Adequate magnification for the surgeon and staff
- Proper understanding of the angle of the hair below the surface of the skin; in almost all instances, the angle of the emergent hair is more acute than the angle of follicle in the dermis. The incision must obviously anticipate this and be oriented in the direction of the follicle rather than the visible hair.
- Punch size of 0.6–1.0 mm in diameter. This size is large enough to encompass the width of the follicular unit, yet small enough to minimise wound size and scarring. Some surgeons have now started using punches of lesser size starting from 0.6 mm.
- Proper motion of the hand: The hand should be perfectly stable while doing short twisting motion of the punch. Bernstein[14] advocates that clockwise rotation (for the right-handed person) generally provides more stability than twisting in the other direction. A back-and-forth motion causes unnecessary transection and is incompatible with successful FUE, as is a 360 degrees rotation of the punch. In some FOX grade 1 cases, direct pressure alone (without any twisting) may be sufficient to extract the grafts.
- Sharp punches/blunt punches: Some surgeons use sharp punch in two-step technique to minimise the amount of twisting needed to cut into the dermis, whereas blunt punches are used in a three-step technique to decrease the follicular transection rate.
- Positive FOX Test

## FOX TEST

It is important to note that the tightness with which follicular units are held in dermis varies and hence FUE may not be suitable in all patients. Therefore, before undertaking any patient for FUE hair transplant, the surgeon should ascertain whether the patient is a suitable candidate for FUE or not. In FOX test, the surgeon takes out a few (about 100) grafts from the donor area and then evaluates how many complete/incomplete follicular units are extracted. If the extraction is easy and complete units are extracted, then the surgeon should go ahead with FUE; otherwise shift onto strip technique.

According to the ease and completeness of extracted grafts, Bernstein and Rassman[14] classified FOX test into five grades. Grade 1 is when intact follicular units literally pop out of the scalp or when there is only occasional transection of individual hairs in the unit. In Fox grade 2 patients, extraction may be relatively easy in the first session, but in subsequent procedures (when the donor area is slightly scarred) it becomes more problematic and the yield starts to decline. In these patients, the longterm yield can be compromised and planning extremely difficult. In FOX grade 3, the emergent angle is difficult. Rassman and Bernstein enrolled 200 patients in a study to assess their candidacy for FUE.[14] They found that 74% of all the patients were either FOX 1, FOX 2 or FOX 3. The description for each category was vague and allows for considerable individual physician discretion and interpretation. In Fox grade 4-5 (when it is almost impossible to predict the emergent angle), the yield is too low for the FUE procedure to be successful. Here, the decision not to use FUE should be straightforward as the transection rate would be too high. If the patient is FOX-positive (grade 1–3), the surgeon can go ahead with FUE in the indications below mentioned.

## INDICATIONS FOR FUE

Following are the indications for FUE:[10]

**Figure 2:**
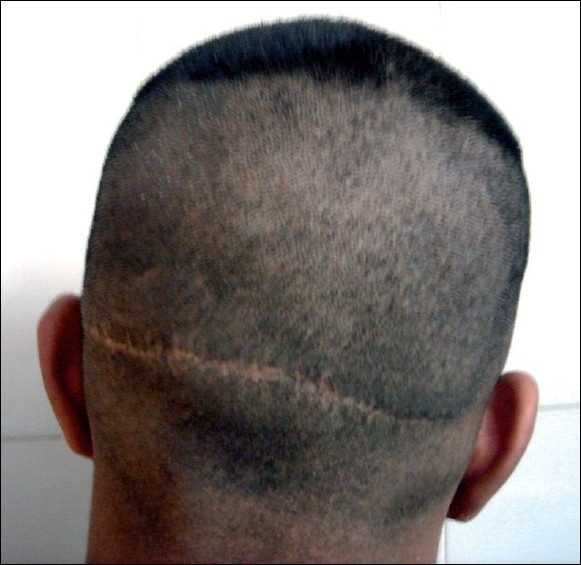
Donor area after strip surgery

1. Patients who want to wear their hair very short (and hence very thin linear scar is unacceptable)
2. When a patient specifically requests an FUE procedure and enough grafts can be harvested to meet his or her needs.
3. In patients with limited hair loss or those who require small sessions. This group includes patients with androgenetic alopecia in Norwood class 3 pattern or small vertex balding areas, limited cosmetic areas such as widow’s peaks (a triangular area of hairloss usually seen in the front of forehead in women), eyebrows, eyelashes, moustaches and limited areas of alopecia secondary to dermatologic conditions.
4. In the treatment of widened scars resulting from traditional strip excisions [Figure 2].
5. Patients having inadequate laxity for a strip excision (too tight skin).
6. For scarring from dermatologic conditions, trauma or neurosurgical procedures.
7. When previous scars of strip surgeries make further strips impossible, then FUE is an indication for further extractions.
8. In patients, who tend to heal with wide or thickened linear scars.
9. In athletes, who must resume full activity immediately after the procedure.
10. For patients with an inordinate fear of pain or scars.
11. When body or beard serves as a donor area.
12. FUE technique is the only technique useful in body hair transplantation. The earlier indication of limited areas of donor site has been overcome by Body Hair Transplant.

## CONTRAINDICATIONS FOR FUE

Following are the contraindications for FUE:[10]

1. Inexperience in performing FUE techniques
2. Unavailability of proper instrumentation
3. Unrealistic patient expectations
4. Inadequate donor supply
5. Scarring that makes both the two- and three-step procedures problematic
6. FOX grade 4 and 5 categories
7. Patient who is not willing for long sessions for several hours or multiple sessions as needed due to the slowness of the process
8. Patient who is not willing to cut his hair short and for this reason women are not good candidates for FUE
9. Large bald areas needing more than 2500 grafts

## PROCEDURE OF FUE

As clarified earlier, FUE is a type of hair transplantation in which the method of extraction is different but implantation is the same as FUT. It is a sutureless method of hair restoration in which hair follicles are extracted from the back of head under local anaesthesia with the help of special micropunches and implanted in the bald area.

On the day of surgery, the entire donor area from the back of the head is trimmed to 1–2 mm length. The patient lies in the prone position on the operating table. Local anaesthesia with Xylocaine, 1% diluted with saline, is administered slowly over the entire donor area.

**Figure 3:**
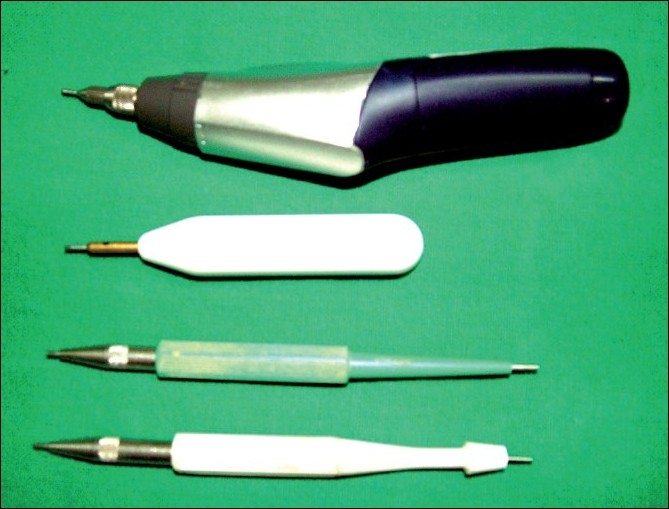
Instruments used in FUE hair transplant

The grafts are then extracted from the donor area with the help of 0.8 and 1 mm special micropunches [Figure 3] The extraction of follicles is done under 2.5 – 5× magnification.

**Step 1:** With the sharp side of the micropunch, scoring of the scalp skin containing follicular unit is done.

**Step 2:** Then dull side of the punch is introduced in the same area and is twisted to loosen the follicular unit. At the same time, the assistant applies counter traction to facilitate the penetration of the punch inside the dermis.

**Step 3:** The assistant gently takes out the graft with the help of forceps. The extracted grafts are then preserved in saline or cool Ringer’s lactate solution.[15,16]

**Figure 4:**
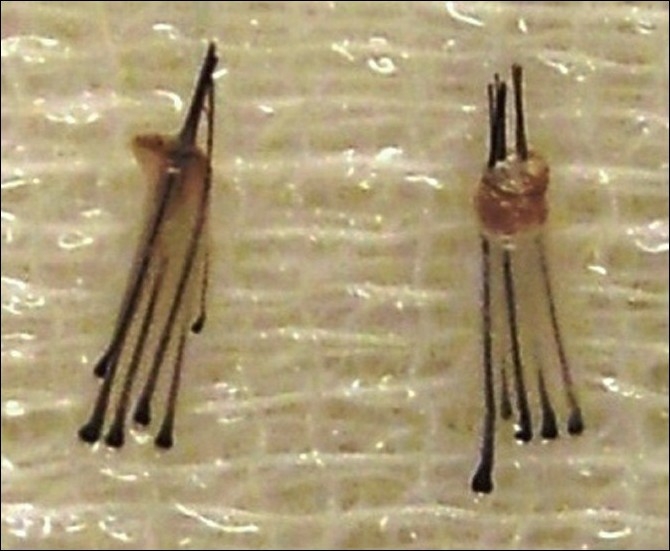
FUE grafts containing 5 and 6 hairs

The extracted graft may consist of 1 to 4 or rarely even 5 or 6 hairs [Figure 4]. This is the most time consuming and tedious part of the whole procedure. After the extraction is over, the grafts are implanted in a similar way as in the rest of FUT.

## EXTRACTION OF GRAFT

There are different ways of extracting the grafts:

### Two-step procedure

There are two steps involved. In the first step, a sharp punch is placed over the follicular unit and aligned according to the direction of the hair shaft. The punch must not be pushed too deep as root transection can occur. In the second step, fine-toothed forceps are used to apply gentle traction to the top of Follicular Unit (FU) until the unit is pulled loose from deeper dermal and subcutaneous connections. There is a person-to-person variation in this technique.[14]

### Three-step procedure

The three-step technique for FUE is based upon Harris’[17,18] concept of using a blunt instrument to prevent damage to follicles during the process of separating the follicular unit from the surrounding donor tissue. He uses the sharp punch to score the epidermis, and then with dull side of the punch bluntly dissects the follicular unit with twisting movement from the surrounding epidermis and dermis. Lastly, the graft is held with forceps and pulled out. In this variation, the dull punch avoids follicle transection and allows intact FUs to be extracted more easily. He found that the graft yield increased from 92% by two-step technique to 98%; and the hair yield from 74 to 93% by the three-step technique.

Although this three-step procedure is superior to the two-step procedure[19] in avoiding follicular transection and in preserving follicular units, there is a greater incidence of buried grafts and is slower. Following measures help reduce or rectify the buried grafts:

- The incidence of buried grafts can be reduced by avoiding the nuchal area (the lower part of the scalp) where the angle of the hair is very acute and the skin has more resistance to the punch.
- Another trick is to clip the hair very short (less than 1 mm) before extracting, as a trapped hair will push the graft deeper into the scalp.
- One can also make the sharp cut slightly deeper and the dull dissection more superficial.
- On occasion, changing the angle of both the sharp and/or blunt instrument can minimise the incidence of buried grafts.
- Sometimes the surgeon may need to revert back to the two-step procedure in select hair transplant patients, particularly those with very coarse hair.
- Finally, one can optimise the blunt-tip design to get the best results.[18]
- In case the surgeon encounters buried grafts, these can be left alone but they may develop into cysts, which may eventually need to be removed.
- If they are not completely buried, the grafts can sometimes be extracted using a small instrument called a Schamberg comedone extractor (the instrument used by dermatologists to remove blackheads).
- The skin may be incised slightly so that the buried graft can be grasped with forceps.

Removing these grafts, although not difficult, is extremely time consuming. If one has a buried graft rate over a fraction of a percent, it becomes a significant logistical problem for the hair transplant surgery and it may result in cyst formation.

## ADVANCEMENTS IN FUE

In the field of FUE, there have been a number of advancements; following is a brief description of the same.

### Follicular isolation technique

The term ‘Follicular Isolation Technique’ (FIT) was coined by Cole and Rose[13] and refers to FUE technique that uses a punch with a ‘stop’ to limit the depth of penetration. Although the need for a depth stop in the extraction technique is still a contentious issue, FIT is possibly a better term than FUE if the entire unit is not being captured. In our view, when the goal is just to extract hair, rather than intact follicular units, the term FIT is preferable.

### Automated FUE hair transplantation

The FUE Matic machine is an automated hair transplant machine that seeks to assist the doctor in performing a hair transplant using the FUE technique.[20,21] It is claimed to give a faster extraction rate of grafts in a limited time. However, there is greater pulling and twisting of grafts which puts the graft at risk of damage, resulting in greater transection.

### Robotics in hair transplantation

Robots have a number of advantages and often enhance and extend human capabilities.[22] Their accuracy and repeatability may reach the sub-millimetre level. Robots can be optimised to perform tasks demanding a high amount of precision at fast speeds, automatically and tirelessly, thus increasing productivity and efficiency. Their performance output is consistent and predictable. These technical strengths may make them suitable for a number of hair transplantation tasks, such as FUE. Some of the drawbacks to robots include cost, non-versatility, inability to process qualitative information and lack of judgement. Efforts are underway to devise such robotics for hair transplantation and it is hoped that they will be available for mass use in the near future.

## ADVANTAGES OF FUE

### Surgeon’s perspective

- It needs less manpower than FUT; One doctor with one or two assistants can run a centre.
- The procedure is less traumatic and surgical experience is not essential.
- Graft preparation is minimal.
- Less equipment is needed.

### Patient’s perspective

- Can sport short hair
- Minimal post-operative recovery time
- Microscopic scars in donor area are almost invisible
- No need to visit surgeon again for stitch removal
- Can use body hair for added density with this technique only
- Can cover preexisting scar of strip surgery with FUE[23]

## LIMITATIONS

1. FUE is a tedious procedure that takes its toll on the surgeon’s patience, energy levels, neck muscles and enthusiasm. Anderson[13] has advised to take short breaks, frequently adjust the posture and to use the assistants well.
2. There is a long learning curve in FUE. Newcomers to this technique find multiple sources of difficulty in performing FUE.
3. Higher transection rate: This remains the main area of concern with this technique. The frequent lack of association between the exit angle of the hair and the subcutaneous course of the follicle is particularly problematic. When this is coupled with frequent changes in follicle direction, the follicular transection rate (FTR) is more.[17] In order to maintain the reliability of FUE, it is indispensable to remain within a permissible level of follicle transection rate (FTR), at least comparable to the standard technique of strip harvesting and microscopic dissection, which has a transection rate of approximately 2%.
4. Tethering of the follicle to dermal components may require either time-consuming dissection or shearing of the follicles as extraction is attempted.
5. The procedure is long and hence tiring for the patient. Patient also has to lie in the prone position which adds to the discomfort.
6. Finally, the number of grafts extracted per day is limited, leading to multiple sessions over several days. To overcome this, surgeons have introduced megasessions. Currently, in some clinics, FUE megasessions up to 2000 grafts over 10–12 hours session in a day are performed. One recent study reports extracting up to 4400 grafts over 3 days.[14]
7. Some surgeons in order to extract higher number of grafts may risk going into the temporary zone; the hair follicles extracted from this region may be lost forever.
8. Very fine trimming of donor hair which is disadvantageous to many people.
9. Only one case can be done in one day. Because of the time spent, the procedure is more costly, almost three times that of FUT.[24,25]

All of these factors have contributed to the relative lack of physicians performing FUE. However, there is much hype on internet sites about this technique and therefore the number of patients seeking the technique is on the rise. Research is needed into the refinement, improvement of instrumentation and efficacy of this technique.[23,24] Table 1 gives a comparison of the strip method and FUE.

**Table 1:** Comparison of FUT strip method and FUE

Observation	Strip	FUE
Pain after the procedure	Minor	None
Percent of time the doctor operates on the patient	10–30%	80–90%
Stitches required	Yes	No
Extensive bleeding during or after the procedure	May occur	No
Wearing hairstyle short in the donor area	Not possible	Possible
Natural results	Yes	Yes
Nerve damage, numbness, permanent pain[25]	Possibly	No
Healing time: donor area	2-3 weeks	Approx. 7 days
Healing time: recipient area	Approx. 14 days	10–14 days
Transection rate (grafts damaged during extraction)	Varies 1–2%	5–10%
Recovery time needed before exercise is possible	2–3 weeks	1–2 weeks
Amount of time after which patient may return to work	The day after	Usually the day after
Visible scarring with short hair at back	May be present	Microscopic scars
Reactions to suture materials	Seen rarely	Never a problem
Shaving of head	not needed	needed
Large areas	possible	difficult
Cost	cheaper	expensive
Fatigue	not tiring	tiring

## CONCLUSION

FUE is an exciting advancement that propels the field of hair transplant surgery one step closer to the elite minimally invasive status. The promise of an almost scarless surgery is enticing to both patient and the surgeon. The reasons for selecting FUE rather than a strip harvest may be the avoidance of a linear scar, the desire for a naturally pain free post-op period or simply the idea of having a minimally invasive procedure.

The technique can serve as an important alternative to traditional hair transplantation in certain selected patients. More research is needed to render the procedure faster, cut short the surgery time and improve the transection rates, so that it can be adopted in greater number of patients.
